# Equestrian Road Safety in the United Kingdom: Factors Associated with Collisions and Horse Fatalities

**DOI:** 10.3390/ani10122403

**Published:** 2020-12-15

**Authors:** Danica Pollard, John Duncan Grewar

**Affiliations:** 1 The British Horse Society, Abbey Park, Stareton, Kenilworth, Warwickshire CV8 2XZ, UK; 2 jDATA Pty (Ltd.), Tambali Village, Sandbaai 7200, South Africa; info@jdata.co.za

**Keywords:** accident, near-miss, vehicle, injury, logistic regression, high visibility clothing, road user

## Abstract

**Simple Summary:**

Horse riders report they frequently experience incidents with other road users, including dangerous near-misses and accidents. The British Horse Society has been collecting information about horse-related road incidents via their website since 2010. The aim of this study was to describe the incidents reported, how they have changed over time and across different UK regions, and the factors which may increase or reduce the risk of collision incidents and those resulting in horses being killed. Road rage and speeding were reported in 40% of incidents while drivers passing the horse too closely were reported in over 80% of incidents. Close passing distance, alone or when combined with speeding, contributed significantly to collisions while speeding alone contributed significantly to horse deaths. Wearing high visibility clothing reduced the risk of having a collision. A horse death caused by a road accident was almost 12 times as likely to result in severe to fatal injury to the rider/handler. Loose horses were more likely to be killed than ridden horses or those pulling a horse-drawn vehicle. Driver behaviour of how to pass horses safely on UK roads needs further improvement and will help reduce the risk of collisions and horse and human fatalities.

**Abstract:**

Over 60% of UK horse riders report having experienced a road-related near-miss or accident. The aim of this study was to describe horse-related road incidents (*n* = 4107) reported to the British Horse Society (2010–2020) and to identify factors associated with higher odds of collisions with another vehicle and horse fatalities using multivariable logistic regression modelling. Drivers passed the horse too closely in 84.2% of incidents while road rage and speeding were reported in 40.3% and 40.1% of incidents, respectively. Close passing distance alone (odds ratio [OR] 18.3, 95% confidence interval [CI] 6.5, 51.6) or in combination with speeding (OR 4.4, CI 1.7, 11.7) was associated with higher collision odds compared to speeding alone. Speeding was, however, associated with higher horse fatality odds (OR 2.3, CI 1.2, 4.6). Wearing high visibility clothing reduced odds of collision (OR 0.2, CI 0.1, 0.4). A fatal injury to a horse was almost 12 times as likely to result in severe to fatal rider/handler injury. Loose horses contribute significantly to road-related horse fatalities. Driver behaviour of how to pass horses safely on UK roads needs further improvement and will help reduce the risk of collisions and horse and human fatalities.

## 1. Introduction

The 2019 National Equestrian Survey estimated that there are 27 million people in Great Britain (GB) with an interest in the equestrian industry with the scale of annual spending in the equestrian sector totalling £4.7 billion [1]. An estimated 1.8 million people are regular riders (riding at least once a month; an increase from 1.3 million in 2015) and 3 million people had ridden a horse at least once in the past year (an increase from 2.7 million in 2015). Interacting with horses and taking part in equestrian activities, although not without its risks [2], has far-reaching physical, psychological and social health benefits [3,4]. The majority of horse riders (94.0% of 428 riders interviewed) in GB take part in pleasure riding activities [1]. Hacking, a term used to describe exercising a horse using a range of equestrian off-road routes and/or public roads, is an integral part of pleasure riding. A survey of 797 randomly selected horse owners in GB between 2009 and 2011 identified that 50.7% had participated in hacking activities in the previous week, hacking a median of two days per week and for a median duration of three hours per week [5]. This indicates that a considerable proportion of the United Kingdom (UK) equestrian population may use public roads to exercise their horses at some point in time.

The extent of road use will likely depend on the availability of designated off-road routes for horses. In fact, 54 infrequent riders (those riding less than once a month) interviewed in 2019, reported that access to safe off-road riding routes was the single main factor preventing them from riding more regularly [1]. Public off-road routes in the UK consist of footpaths (useable only by pedestrians, mobility scooters and powered wheelchairs), bridleways (multiuser routes for footpath user groups and including cyclists and horse riders), restricted byways (multiuser routes for footpath and bridleway user groups, including horse-drawn vehicles) and byways open to all traffic [6]. Horse-drawn vehicles have even further restrictions with regards to access to off-road routes, being only permitted on public roads and byways but not bridleways. Even when there is a good network of equestrian off-road routes in the area, road use is often required to travel to, and/or between, them. Permissive or private off-road routes are provided at the landowners’ discretion for different user groups but access to these may be revoked at any time. In areas with a low frequency of public equestrian off-road routes, permissive routes may be restricted seasonally or be tolled (requiring a subscription fee to use) and road use may be the main exercise option.

A study of cyclist road safety in urban settings identified that better safety outcomes were associated with a greater prevalence of bike facilities, such as protected and separated cycling paths which removed cyclists from the road, and lower-speed environments [7]. With the rapid development and urbanisation of the UK’s countryside, little priority is placed on creating new equestrian off-road routes and the 2026 deadline for reinstating historic bridleways is fast approaching. Public access will be lost to bridleways not formally recorded by 1st January 2026 [8]. Fewer off-road options for equestrians increase the potential for dangerous interactions between them and other road users. Additionally, most rural single-lane roads in the UK have speed limits of 60 miles per hour (mph) (~97 km per hour [km/h]), placing equestrians at high risk of dangerous interactions with motorised vehicles on narrow roads with often poor visibility.

The Department for Transport (DfT) police-reported road accident database shows that in 2019, out of 124 people involved in road accidents including ridden horses, 55.6% (*n* = 69) were injured and of those injured, 94.2% (*n* = 65/69) were horse riders [9]. However, the DfT figures represent only ridden horses, excluding accidents involving non-ridden horses such as horse-drawn vehicles, and a small fraction of actual injury-causing incidents. Hospital Episode Statistics for England alone document that between April 2019 and April 2020, 3298 people were admitted to hospital due to an animal-rider or animal-drawn vehicle transport accident, with 89.1% (*n* = 2940) being emergency admissions [10]. Several campaigns have been launched to raise awareness of the vulnerability of equestrians on the road [11,12,13] and the increased popularity of helmet and dashboard cameras has provided some alarming examples of dangerous interactions between equestrians and other road users. A survey of 426 UK horse riders revealed 60.3% reported having a dangerous near-miss on the road in the previous year [14], which is similar to 52.0% of 147 Australian riders that experienced at least one accident or near-miss in the past year [15]. A study in Devon County found that 79.1% (1563/1976) of riders sampled reported they had previously experienced a near-miss [16]. Apart from the obvious life-threatening or life-altering damage a human or horse may suffer in the aftermath of a road incident, the psychological trauma experienced by both may substantially alter their behaviour, even when no serious physical injury occurs [17,18]. Negative experiences and perceptions of other road users could increase anxiety in both the horse and rider, deterring them from using certain routes or roads altogether. While road safety relies on the responsible behaviour of all, those with the potential to do more harm should take greater care when interacting with more vulnerable road users. Despite the far-reaching impact equestrian road safety has in the UK, it is a relatively under-researched and under-funded topic [19]. Highlighting the challenges faced by equestrians when using public roads in the UK is of great importance.

In the current study, the focus is on collisions and horse-related injury outcomes. The aims of this study are to:

(a) describe the types of incidents reported to the British Horse Society (BHS) Horse Incidents website [20] between January 2010 and September 2020 and the circumstances surrounding them
(b) identify factors associated with higher odds of incidents involving collisions between a horse, rider/handler or horse-drawn vehicle and another road user
(c) identify factors associated with higher odds of incidents resulting in horse fatality.

## 2. Materials and Methods

The BHS has been collecting horse-related incident data via online reporting forms on the Horse Incidents website since 2010 [21]. Incident types that can be reported include a variety of on- and off-road incidents including other road or off-road users, equine transport, dogs, fireworks, bird scarers, slippery road surfaces, low flying aircraft and drones. Each incident type has a purpose-designed form. Incidents are self-reported and can be submitted by anyone directly involved in, or having witnessed, a horse-related incident. The Road Incident Form (Supplementary information, Form S1) consists of a combination of closed- and open-ended questions. The form collects information about the incident, including:

- incident date (day, month and year), time (hour and minutes) and location (latitude and longitude coordinates and region);
- the reporter’s details and their involvement in the incident (rider/handler, motorist, witness, friend, police or other);
- incident circumstances including the type of road, speed limit (if known), road surface conditions, area type and visibility conditions;
- behaviour of road users including whether road rage was directed at the equestrian by the other road user, loss of horse or vehicle control, whether the vehicle was exceeding the speed limit (as perceived by the reporter) or passing the horse too closely and whether a collision occurred between the vehicle and the horse;
- details on the main horse and rider/handler involved in the incident, including the subjectively-assessed severity of any injuries (none, mild, moderate, severe, fatal), the status/use of the horse at the time (ridden, pulling a horse-drawn vehicle, being led by a person on foot, loose [absence of any human handler] on the road), as well as whether any safety equipment was worn by the equestrian, such as high visibility clothing, a riding helmet and body protector;
- any other details relating to the incident in free text boxes.

Although originally called the Horse Accidents website, the website was renamed to the Horse Incidents website and the BHS has been encouraging equestrians to report both incidents resulting in injury and near-misses which had the potential to cause injury. In 2016, the BHS launched the “Dead? or Dead Slow?” campaign [11] to provide road safety education and advice to both vehicle drivers and equestrian road users. The campaign focuses on educating vehicle drivers to pass horses they encounter on roads wide (2 m distance) and slow (maximum of 15 mph or 24 km/h).

### Data Analysis

Road-related incidents occurring over an almost 10-year period (between January 2010 and September 2020) were downloaded from the BHS database into a Microsoft Excel (Office 365, Microsoft Corporation, Redmond, Washington, USA) spreadsheet and imported into statistical software Stata (IC v. 13.0, StataCorp LP, College Station, TX, USA) for cleaning, coding and statistical analysis. Incident reports occurring on the same date and at the same geographical location were reviewed to exclude duplicate reports. Incidents not related to road incidents were excluded. For incident time, minutes were rounded down to the nearest hour to allow creation of time categories. A single person could report multiple incidents and reporter details were used to match reports belonging to the same person via a reporter ID variable. Data were subsequently anonymised for further analyses. Normality of distribution for continuous variables was assessed formally with the Shapiro-Wilk test and visually with histograms and overlaid normal and kernel density plots. Continuous and ordinal variables were described as medians with interquartile range (IQR) and range, and categorical variables were described as proportions (%) with 95% confidence intervals (CI).

Regarding collisions, the dedicated question within the reporting form asked specifically whether a collision had occurred between a horse and a person in charge of a vehicle (such as car, van, lorry or bus drivers, drivers of agricultural vehicles such as tractors, motorcyclists and cyclists). Open-ended incident description text was reviewed to identify incidents where collisions had occurred between another vehicle and a rider/handler or a horse-drawn vehicle, but not necessarily the horse. A binary outcome variable was created to represent whether the reported incident involved a collision (1) or did not (0). Collisions were, therefore, defined as any unintended physical contact between a horse, rider or handler and/or horse-drawn vehicle and a person in charge of a vehicle on a public road. For example, this included vehicles hitting the rider in the leg when travelling past or hitting a handler of the horse if the horse was being led from the ground.

A second binary outcome variable was created to differentiate between horse-related road incidents resulting in a horse fatality (1) and incidents not resulting in a horse fatality at the time of reporting (0). Continuous variables were assessed for evidence against linearity (likelihood ratio statistic [LRS] *p*-value < 0.05) by recoding initially into quartile categories, followed by recoding into biologically plausible categories. The spatial distribution of the total number of incidents and the proportion of incidents involving collisions that occurred between 2010 and 2020, and divided between two time periods (2010 to 2015 and 2016 to 2020) were mapped using reported latitude and longitude coordinates. Choropleth maps were produced with incident counts aggregated onto a hexagon grid overlaying the UK. Hexagons were ~33.5 km (20.8 miles) on their longest diagonal and ~722 km^2^ (279 miles^2^) in area. Exploratory cluster analysis was performed using SaTScan (v9.6.1, Kulldorff, M and Information Management Services, Inc. 2009. SaTScanTM v8.0: Software for the spatial and space-time scan statistics. htpp://www.satscan.org). Specifically, a retrospective space-time analysis scanning for clusters with high rates using the space-time permutation model was applied [22]. Cluster radius was limited to a maximum of 50 km (31 miles) with time aggregation set at a single month. Clusters were further filtered to include only those with a *p*-value of < 0.05. All mapping was produced in QGIS 3.10 (http://qgis.org). Missing data remained missing so that individual reports with missing data on variables of interest were automatically excluded from the analyses. The only exception being 38 records where the day the incident occurred was not reported. For mapping and cluster analysis purposes, these records were assigned to the 15th of the month and year reported.

Initial relationships between the collision and horse fatality outcomes and potential explanatory variables were assessed using the Chi-square test or Fisher’s exact test for categorical data and the Mann-Whitney *U* test for continuous data. Univariable mixed-effects logistic regression modelling was used to identify factors associated with higher odds of incidents involving a collision by calculating odds ratios (OR) and corresponding 95% CIs with reporter ID as a random effect to account for repeated observations by the same reporter. Variables where LRS *p* < 0.25 were taken forward to multivariable modelling. Manual, forward selection was used to create the final multivariable mixed-effects logistic regression model, with stepwise addition of variables from most to least significant based on their LRS *p*-values. Variables were retained in the final model if they significantly improved model fit (LRS *p* < 0.05). All variables not retained in the final model were individually forced back into the model in order to assess any potential interactions or confounders.

An ordinary logistic regression model was used to identify factors associated with higher odds of incidents involving a horse fatality. A random effect for reporter ID was not included as it was considered unlikely that the same person would be involved in more than one horse fatality incident. The final multivariable logistic regression model was built in the same way as described above. The Hosmer–Lemeshow goodness-of-fit test was used to assess the fit of the final logistic regression models, excluding random effects, to the data [23]. Additionally, a receiver operating characteristic (ROC) curve was estimated to calculate the area under the curve.

## 3. Results

A total of 4140 horse-related road incidents were reported to the BHS between January 2010 and September 2020. Thirty-three incidents were excluded (25 duplicate incidents, 6 not located in UK and 2 related to horses being transported in a trailer/horsebox). Therefore, a total of 4107 incidents remained available for descriptive and statistical analyses. Table 1 provides a summary of the road incident data collected. Due to missing data, the denominator for each variable may vary.

### 3.1. Incident Details

The highest frequency of incidents was reported in the spring and summer months, accounting for 56.9% (*n* = 2330) of the incidents reported. The majority of reported incidents (54.9%, *n* = 2187) occurred in the time period between 10:00 and 14:00. There has been a steady increase in the number of incidents reported each year since 2010, with 2019 (the last full year on record) having the highest frequency of incidents reported. Less than half of the incidents (42.4%, *n* = 1708) were also reported to the police at the time of reporting to the BHS, although some reporters said they would be contacting the police in the future.

**Figure 1:**
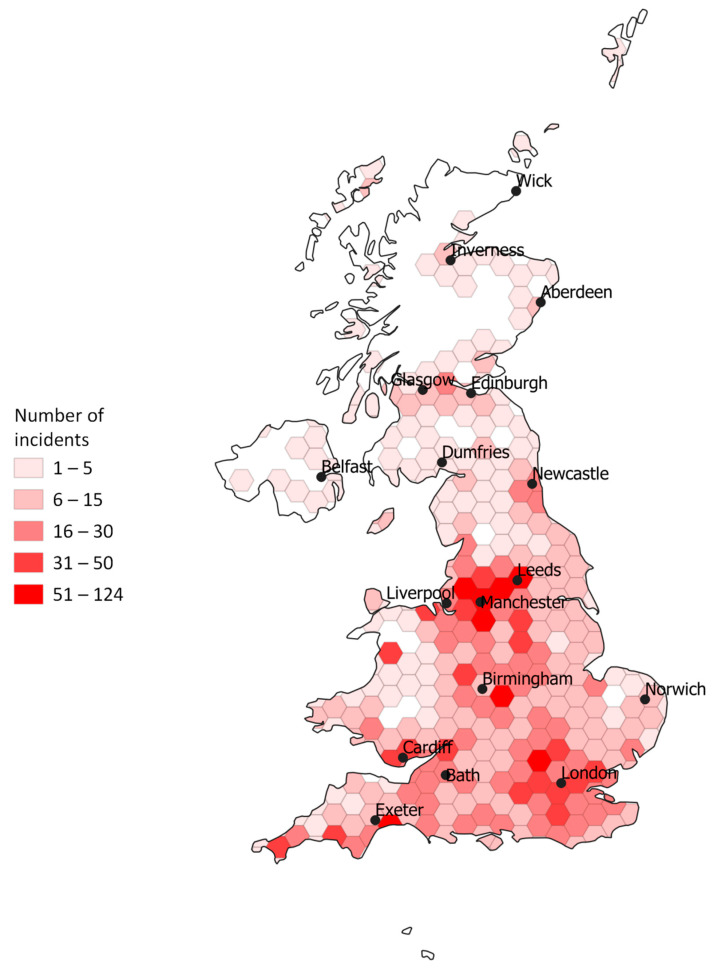
Choropleth map of all horse-related road incidents reported to the British Horse Society across the United Kingdom (UK) between 2010 and 2020. Major UK cities and towns are labelled.

**Figure 2:**
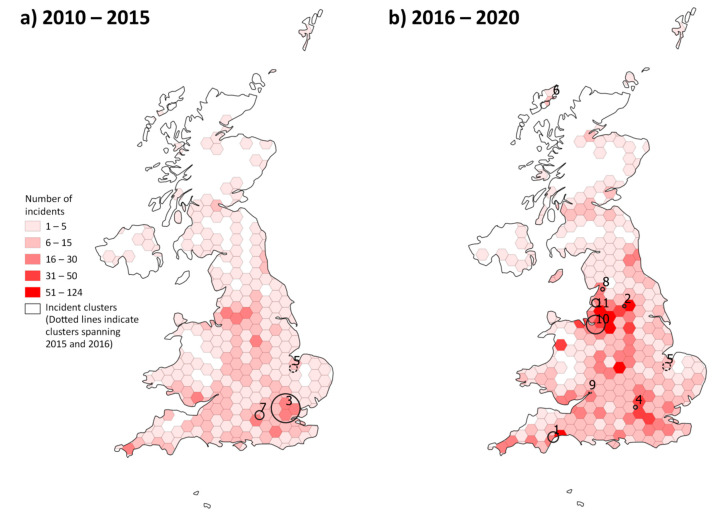
Choropleth map of horse-related road incidents reported to the British Horse Society across the United Kingdom divided across two time periods (**a**) 2010–2015 and (**b**) 2016–2020. Significant clusters (*n* = 11) are depicted by solid/dotted circles and are labelled by their ID referenced in Supplementary information, Table S2. Cluster #5 which spans 2015/2016 is depicted by a dotted circle.

The regions of the UK with the highest frequency of reported incidents were the South West (16.8%, *n* = 691), South East (15.1%, *n* = 620) and West Midlands (14.0%, *n* = 574). Due to missing latitude and longitude and/or day and month data for some of the incidents, 4070 incidents were available for mapping and exploratory cluster analysis (Figure 1 and Figure 2). There were a total of 11 significant clusters detected over the 10-year period (Supplementary information, Table S2). The space-time permutation cluster model effectively evaluates incidents where clusters are identified if, during a specific time period and within a geographic area, more incidents are proportionally present compared to the remaining geographical area encompassing the dataset. Clusters that are significant contain both a spatial and time component – hence the space-time permutation model.

**Figure 3:**
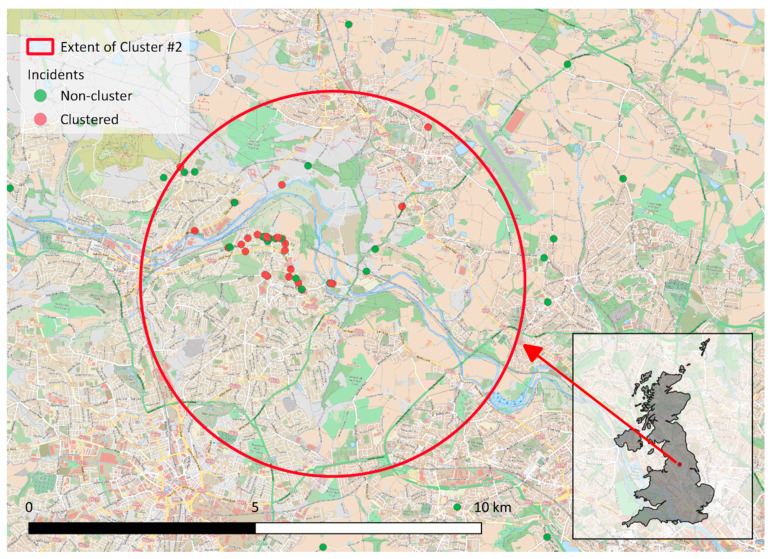
Cluster #2, near Leeds in Yorkshire, depicting the spatial component by the large red circle and the time component by those points that were part of the cluster in red (incidents occurred between 3 Aug 2016 until 2 June 2017) while those that are not considered clustered in green. The inset map depicts the location of Cluster #2 in relation to the United Kingdom – also refer to Figure 2b. The underlying background of the main map is the Wikipedia Bike Hike Map which is hosted by OpenStreetMap (https://www.openstreetmap.org) and available under a CC BY-SA 3.0 license (https://wiki.osmfoundation.org/wiki/Terms_of_Use).

Figure 3 depicts cluster #2 and is an example of the cluster outcome. In Figure 3 there are incidents (red points) that have been identified as potentially clustering in that space and time (between 3 August 2016 and 2 June 2017). Note the incidents (green points) that occur in the same area but in relation to all other incidents did not occur at a proportion that was significantly higher than other incidents in the UK.

Spatially, clusters generally mimicked underlying incident proportions and most (*n* = 9) were identified between 2016–2020, with one of those spanning 2015/2016 (cluster #5). Clusters had a mean and median radius of 11km (6.8 miles) and 9.9km (6.2 miles) respectively with a range of 0.27 to 36.63 km (0.2 to 22.8 miles). Temporally, clusters occurred over a wide range of periods with a mean and median of 234 days and 91 days respectively and ranging between 29 and 942 days.

Most of the incidents occurred on minor (48.4%, *n* = 1956) and secondary (20.1%, *n* = 813) roads and in rural areas (74.4%, *n* = 2963). The highest frequency of incidents (32.4%, *n* = 1331) were reported to occur on roads with speed limits of 60 mph, although a considerable proportion of speed limits were not known (20.5%, *n* = 841). Weather conditions during most incidents were dry (73.5%, *n* = 2864) with good visibility (90.9%, *n* = 3503). Most road surfaces where incidents were reported were worn (72.1%, *n* = 2739). Road rage by vehicle drivers was reported in 40.3% (*n* = 1579) of incidents. The majority of incidents involved a driver of the vehicle passing too close to the horse (84.2%, *n*=3325) and a smaller proportion of incidents involved the driver of the vehicle exceeding the speed limit (40.1%, *n* = 1541). When the passing distance and speed variables were combined, 34.8% (*n* = 1323) of vehicle drivers were reported to exceed the speed limit while also passing the horse too closely. While only a small proportion of vehicle drivers were reported to have lost control of the vehicle during the incident (9.3%, *n* = 360), 33.2% (*n* = 1270) of riders/handlers were reported to have lost control of the horse.

### 3.2. Details of the Main Horse Involved in the Incident

The median age of the main horses involved in the incident was 11 years (IQR 7, 15; range 1, 32 years) and most of the horses (73.3%, *n* = 2846) used roads regularly (more than once per week). Almost 90.0% (*n* = 3681) of incidents involved ridden horses, although horses being led, driven in a horse-drawn vehicle and loose on the road were also represented. For example, one reporter commented: “*I was leading my horse along the road from field to stable when both me and my horse were hit by a Ford focus from behind I was thrown over the top of the car resulting in spinal fracture, bleed on the brain, whiplash, ligament tendon and cartridge damage to knees and legs and a frozen shoulder my horse was hit down her right side with the wing mirror multiple witnesses police, ambulance and air ambulance attended the seen the police claimed the driver did not see us due to his dirty windows screen and the sun was in his eyes he failed to reduce his speed…[sic]”*

Although most horses were not reported to have physical injuries, 18.6% (*n* = 762) had mild to moderate injuries and 3.2% (*n* = 130) died or were euthanased as a result of the injuries sustained. However, several riders/handlers commented that the incident had a psychological impact on their horse:

[Reporter 1] *“Car approaching from front going to fast. I asked her to slow down - she braked hard and skidded on loose gravel on the road. Terrifeid the horse who spun around in front of the car and galloped down the road. We were so lucky that nothing was coming up fast behind us or I would not be here to write this note. Haven’t taken this horse on the road since because it scared her so much (and me).”*

*[sic]*

[Reporter 2] *“Vehicle came up behind horse too close. Rider waved driver on to pass when safe to do so but obviously driver was annoyed at being delayed for a few seconds so dropped car into 2nd gear and sped past startling the horse who then ended up in a ditch, scrabbled back out and nearly ended up on another car in his frightened state. Fortunately, rider is very experianced and was able to stay on and regain control BUT rider and horse were both very badly shaken and this is a horse who has never had a problem hacking out before and has been along this route frequently for 3 years.”*

*[sic]*

[Reporter 3] *“Tractor driver scared horses by scraping sprayer arms on hedging on a single track road, my horse and husbands horse ended up down the dyke. Tractor driver continued even after seeing both of us in a dyke and after my horse ran along the dyke bottom then back out onto the road in front of him and husband fell off in a ploughed field. Tractor driver then refused to turn off engine even after my horse cantering on the spot terrified and husbands horse loose and now cantering along the road. He had to stop eventually as my horse was blocking the road and the loose horse was on the road…he then revved his engine and lunged towards us before I could catch hold of the loose horses’ rein, he then proceeded to chase the loose horse onto a main A road into all the traffic.* *We now have 2 horses that were previously excellent with tractors now nervous…”*

*[sic]*

[Reporter 4] *“Two of us riding (17.2hh and 15.2hh both skewbald easy to spot!) single file, at walk, wearing Hi viz, an oil tanker came very fast around a double bend. I was behind the first horse and started to wave them down to slow up. There was no attempt to slow down and as the lorry came closer (huge and loud) both horses turned tail and bolted away. Still the tanker didn’t slow down and came past us as we were bolting up the road! There was no attempt to slow or acknowledge what was happening, the tanker just carried on up the road and away. Luckily both riders were able to bring their horses back to walk and turn back for home. The 17.2hh horse had always been a very sensible hack but this incident has made him very nervous out on the roads.”*

*[sic]*

**Figure 4:**
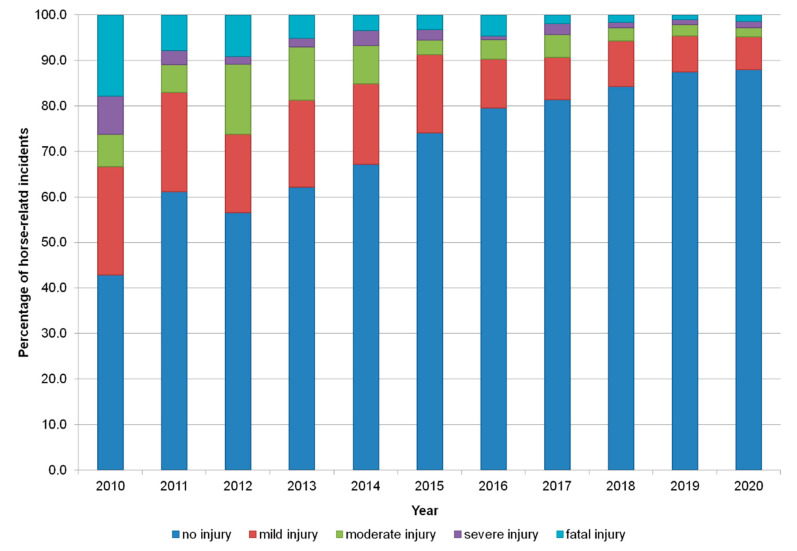
Road incidents (*n* = 4089) between equestrians and vehicle drivers reported to the British Horse Society and injury severity sustained by the horse over an almost 10-year period (2010–September 2020).

**Figure 5:**
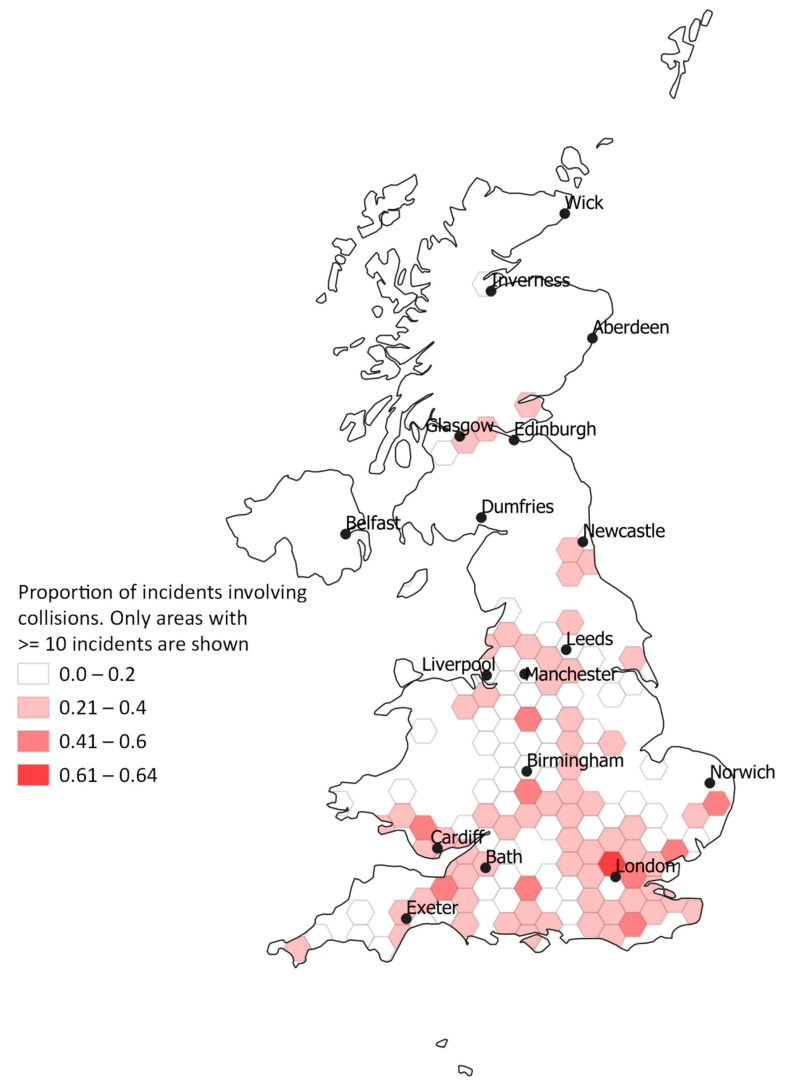
Choropleth map showing the proportion of incidents involving collisions portrayed throughout the United Kingdom where at least 10 incidents per hexagon area took place.

The proportion of reported incidents leading to horse injury have decreased since 2010, where they represented more than 50% (48/84) of the incidents reported to approximately 13% (114/914) in 2019 (Figure 4), showing an increase in the reporting of near-misses over time in the face of an increase in incident reporting (Figure 2). A small proportion (8.6%, *n* = 348) of horses were reported to have received veterinary treatment. Over twenty percent of incidents (*n* = 855) involved a collision with a motorised vehicle and in most collision-related incidents the point of impact was from the rear (63.0%, *n* = 486/771). Incidents involving collisions occurred less than expected in regions surrounding Liverpool, Manchester and Leeds (North West and West Yorkshire) when compared to the map of all incidents (Figure 5). However, the proportion of collision-related incidents did appear to be higher in the proximity of large cities, and particularly in England.

### 3.3. Details of the Main Rider/Handler Involved in the Incident

The median age of the main rider/handler involved in the incident was 40 years (IQR 27, 50; range 5, 81 years) and the majority were female (88.8%, *n* = 3646). Most riders/handlers (72.9%, *n* = 2781) had more than 15 years of equestrian experience on the road and just over a third (35.5%, *n* = 1309) said they had passed the BHS Riding and Road safety test. Rider/handler falls occurred in 16.7% (*n* = 684) of incidents. Similar to the horse injury data, the majority (77.7%, *n* = 3172) of riders/handlers did not sustain injuries, but 21.8% (*n* = 889) sustained mild to severe injuries and 0.5% (*n* = 21) died as a result of the injuries sustained. Most riders/handlers wore safety or protective equipment such as high visibility clothing (91.2%, *n* = 3608) and riding helmets (95.3%, *n* = 3714) but few wore body protectors (2.3%, *n* = 88). Although not always seriously injured, several riders/handlers reported that they, or those around them, were negatively affected by the incident:

[Reporter 6] *“Four off road bikes ridden by young lads two of which purposely revved up behind us which spooked the horse sending him backwards. My daughter managed to grab him before we ended up in a ditch the other two boys obviously scared responded to my yelling at them to slow down and lower their revs. My daughter has now said she will never ride out on the roads again, which saddens me. My horse is just over 20 years old and has and will always be my rock, he is used to traffic including motor cycles, tractors etc. but he was shaking after this, I realise that no one was injured but we are very shook up.“*

*[sic]*

[Reporter 7] *“Driver come around bend on my side of road head on at a horrendous speed well in excess of the speed limit. The driver then skidded his 4x4 which spooked my pony. The pony spun clock wise still on our side of road as the 4x4 collided into us both. The driver then got out vehicle and verbally abused me before fleeing the accident without giving details... Riders are now frightened to ride this fast road since my accident.“*

*[sic]*

[Reporter 8] *“when the tractor driver was asked clearly to wait before pulling onto the road, he ignored and proceeded to pick up speed and after several attempts from my passenger to ask him to stop he continued to move forward causing the horse to spin and panic tipping the cart on its side in a ditch and throwing us out, he then bolted out the ditch along the road with the cart on its side til it corrected itself and another tractor driver behind us had witnessed the incident jumped out of his tractor and caught my horse. the horse is traumatised, both myself and my passenger are sore and bruised and my cart is destroyed.“*

*[sic]*

### 3.4. Details of the Person Reporting the Incident

Most of the incidents were reported by the rider/handler themselves (86.4%, *n* = 3472), although incidents were also reported by other parties, such as witnesses (3.5%, *n* = 142) and motorists (3.5%, *n* = 139). For example, one witness reported: “*Lady on her horse riding on the road. Everyone in front of [driver] was whizzing past the horse and rider, though her horse was handling it well. [Driver] slowed right down and pootled along on the other side of the road past the horse and rider. AS [driver] was passing the horse and rider a man in a black BMW undertook [driver], in between her and the horse and rider. Drove past at top speed and honked his horn when directly in front of horse and rider. [sic]*”

Most of the incident reporters (64.9%, *n* = 2665) did not have a membership with the BHS indicating that knowledge of the Horse Incidents Website has reached outside the organisation.

### 3.5. Factors Associated with Higher Odds of Incidents Involving Vehicle-Related Collisions

The results of the univariable mixed-effects logistic regression modelling are presented in Supplementary information, Table S3. Following multivariable modelling, eight variables were found to be significantly associated with vehicle collisions (Table 2). There was evidence of a lack of independence of observations at the reporter level (*σ* = 2.02, *ρ* = 0.55, LRS < 0.001). The odds of collisions were almost four times as high (OR 3.6, 95% CI 2.5, 5.2) in the earlier year period (2010–2015) compared to the later (2016–2020). Incidents reported in the afternoon or early evening (between 15:00 and 19:00) had higher odds of collisions (OR 1.4, 95% CI 1.1, 2.0) compared to incidents reported between late morning and early afternoon (between 10:00 and 14:00). There were regional differences in odds of collisions; regions with highest odds of collisions, relative to the North West which had the lowest odds, were the South East (OR 2.1, 95% CI 1.2, 3.9) and Northern Ireland (OR 11.7, 95% CI 2.4, 56.8). Odds of collisions were higher on roads with lower reported speed limits (between 20 to 40 mph) compared to roads with speed limits of 60 mph (*p* = 0.002). However, odds of collisions were similarly higher on roads where the speed limits were not known by the reporter (*p* = 0.001). Incidents where road rage was reported, such as abusive language or intimidating behaviour by the vehicle driver, were associated with lower odds of collisions (OR 0.2, 95% CI 0.2, 0.4). Compared to vehicle drivers exceeding the speed limit only, passing the horse too closely, even when not perceived to be speeding, was associated with highest odds of collision (OR 18.3, 95% CI 6.5, 51.6). However, exceeding the speed limit and passing too closely in combination were associated with over four times the odds of collision compared to exceeding the speed limit alone (OR 4.4., 95% CI 1.7, 11.7). There was a linear relationship between the age of the main rider/handler involved in the incident and odds of a collision with the odds decreasing by 0.97 with each increasing year in age (*p* < 0.001). Finally, incidents, where the rider/handler or horse wore high visibility clothing, were associated with lower odds of collisions (OR 0.2, 95% CI 0.1, 0.4); when high visibility clothing was worn there was an 80% decrease in the odds of being involved in a collision compared to a non-collision incident.

The Hosmer–Lemeshow goodness-of-fit test (*p* = 0.53, χ^2^ 3.17) suggested that the ordinary logistic regression model described the data well. The area under the ROC curve was 0.745 (CI 0.72, 0.77), indicating that the model had reasonable over-all predictive power.

### 3.6. Factors Associated with Higher Odds of Incidents Involving Horse Fatality

The results of the univariable logistic regression modelling are presented in Supplementary information, Table S4. Following multivariable modelling, seven variables were found to be significantly associated with incidents resulting in horse fatality (Table 3). There were yearly differences in the odds of horse fatality with odds higher between 2010 and 2013, and 2015 and 2016, compared to 2019 (*p* = 0.002). Similar to the collision data, incidents where road rage was reported were associated with lower odds of horse fatality (OR 0.2, 95% CI 0.1, 0.6). However, vehicle speed was associated with higher odds of horse fatality, with odds of fatality more than doubled when the speed limit was being exceeded (OR 2.3, 95% CI 1.2, 4.6). Unsurprisingly, collisions with vehicles (OR 73.2, 95% CI 17.2, 310.9) and whether the horse lost their footing and fell (OR 5.2, 95% CI 2.6, 10.1) during the incident were associated with higher odds of horse fatality. Incidents involving reports of loose horses (loose for a variety of reasons including having escaped from the rider/handler, their field or being free-roaming ponies in a national park) were more likely to result in a horse fatality (OR 75.0, 95% CI 19.8, 284.0) compared to when the horse was ridden or pulling a horse-drawn vehicle. Lastly, the severity of injuries sustained by the horse and rider/handler were strongly associated, with increasing severity of rider/handler injury associated with higher odds of horse fatality (*p* < 0.001).

The Hosmer–Lemeshow goodness-of-fit test (*p* = 0.94, χ^2^ 0.78) suggested that the ordinary logistic regression model described the data well. The area under the ROC curve was 0.980 (CI 0.97, 0.99), indicating that the model had good over-all predictive power.

## 4. Discussion

This study provides valuable insight into road incidents involving horses reported to the British Horse Society between 2010 and 2020 and highlights the challenges faced by many equestrians when using public roads in the UK. Spatial mapping of the incident and collision data have identified regions of the UK of particular interest, which could signify incident hotspots or clusters. While the causal parameters of clusters were not elucidated, our results indicate that clusters are likely to occur and it would be worthwhile to establish why this is. In the significant clusters alone, approximately 236 incidents occurred over and above the expected rate. Additionally, the data have identified factors which may help to reduce the odds of collisions between vehicles and horses, as well as horse fatalities. This evidence-base could be used to investigate targeted interventions, such as road safety education, improved road signage, and research into specific road-user behaviour change campaigns.

### 4.1. Incident Details

#### 4.1.1. Regional and Seasonal Distribution

It is well-established that road accidents are often under-reported, particularly if they do not result in serious injury or human fatality [24]. Less than half of the incidents reported to the BHS were also reported to the police. The highest frequency of incidents coincided with the times of the year when horse riders report they hack more frequently and cover further distances [16] with 57% of the incidents reported being in the spring and summer months. Similarly, the time of day (late morning to early afternoon), road and area types (minor and secondary road and rural areas) and weather conditions (dry with good visibility) most frequently reported in the incident details coincide with areas where equestrians are more likely to access roads and the environmental conditions in which they do so. For example, equestrians may actively choose not to access roads during morning and afternoon/evening rush-hour periods or during poor weather conditions when visibility may be compromised. The South West, South East and West Midlands regions had the highest frequency of reported incidents, with Greater London having the lowest. These regional reporting frequencies likely represents the distribution of equestrians in the UK [1], awareness of the incident reporting system, and personal motivations for reporting an incident. There were no population at risk or control data available to evaluate clusters where negative (non-incident) occurrences were available, and we, therefore, had few options in terms of model selection for cluster analysis. When performing space-time permutation clustering analysis, there is potential for bias to occur when the period under review is longer than a few years, as was the case in the present analysis. This bias occurs, for example, where a local area’s horse-use changes because of an increase in horse-use population and incidents increase in proportion to this. While clustering is identified here, it does not represent a true cluster, rather one of proportional use. If horse-use population changes homogenously over the study area and throughout the study period, then this bias is not a concern. Further research is required to understand the patterns, and associated reasons, of equestrian activity when using roads with their horses. In particular, their experiences and perceptions of other road users and what motivates them to report an incident.

#### 4.1.2. Interactions with Other Road Users

The main riders/handlers involved in the reported incidents were predominantly females in their 40s with more than 15 years of equestrian road experience, although the age of those involved ranged from as young as five to over 80 years. This reflects the demographics of the underlying equestrian population, with female equestrians in the majority [16] as well as the range of ages that use the roads, including children. Description of the behaviours of other road users around horses on the road paints a worrying picture, suggesting a disconnect with the existence of an extra brain (belonging to the horse) that needs to be taken into consideration. Road rage, such as aggressive or intimidating driving (for example tailgating or deliberately driving at the horse or handler to force them off the road), repeatedly sounding the horn or verbal abuse, was reported in 40% of incidents. This is considerably higher than the 17% of equestrians that reported having experienced abuse (yelling, cursing, intentional chasing, having objects thrown at them or repeatedly ‘beeping’ the horn) by drivers of a car in an Australian study [15]. However, it is likely that equestrians that experienced particularly bad road rage may also be more likely to report the incident. The most common behaviour by other vehicle drivers in this study was not allowing enough space when passing the horse. Less than half of the equestrians reported speeding to have occurred, which was the most cited contributor to near-misses or accidents in interviews with Australian riders [15]. However, it is important to consider the context in which this question was asked: ‘Was the vehicle driver exceeding the speed limit?’ Therefore, the proportion of speeding vehicle drivers in the current study is an underestimation of vehicles that were exceeding the 15 mph speed advised when passing horses on the road, even though they may have been within the legal speed limit. For example, one reporter commented about a vehicle passing them:

*“To close and too fast although not over speed limit. Tried to squeeze between me and a car on the other side of the road that had just passed us.“*

In a 2020 review of the current UK Highway Code, the BHS has proposed inclusion of explicit advice for vehicle drivers on how to pass horses safely on the road, passing widely (at least 2 m distance) and slowly (maximum of 15 mph). However, this message requires further emphasis and dissemination within the driving community as it is likely that few vehicle drivers with existing licenses will read the updated version of the Highway Code.

Several equestrians mentioned that, when engaging with the other vehicle drivers, comments such as “*Horses should not be on roads*” were made. This is in keeping with the Australian study where several participants reported a feeling of being unwelcome or a trespasser on the road due to the actions or words of other vehicle drivers [15]. Questioning the legitimacy of other road users by vehicle drivers is not confined to equestrians alone. Negative vehicle driver attitudes towards cyclists have been associated with the perception that cyclists do not financially contribute to road usage, having no obligation to pay road tax or insurance [25]. However, the majority of equestrians in the UK keep their horses up to 10 miles from their own residence [26] and so are highly likely to also be vehicle drivers. Additionally, 93% of equestrians in the current study said they had existing public liability insurance for road use with their horses. On the other hand, equestrianism may be perceived as an expensive and elitist leisure activity, potentially viewed as ‘upper-class’ if associated with an activity such as fox hunting [27]. Drivers are also more likely to show empathy to equestrians if they themselves have direct or indirect experience with horses [19]. Encountering a slow-moving road user can evoke feelings of frustration, particularly in younger drivers, because of the feeling of holding up an essential or work-related journey [19]. These behaviours by other road users suggest a fundamental misunderstanding of the reasons why equestrians use roads (usually not because they want to but because it is the only way to connect up off-road tracks) as well as the physical and behavioural characteristics of a horse. Horses are prey animals and react and process information very differently to humans [28]. Even the well-trained horse may react unpredictably in a certain situation. Lack of awareness of the typical reaction of a frightened horse, and how quickly and how far they may move when startled, probably plays a large role. The average adult horse weighs approximately 500 kg and is capable of travelling at speeds of 50 to 60 km/h (the equivalent of approximately 30 to 40 mph) [15,29,30]. Horse-riding is recognised as a dangerous sport and this danger is magnified when equestrians have negative interactions with other road users [31,32,33]. Horses may respond to frightening situations by blindly bolting away from the frightening stimulus or stepping away from it into the path of surrounding traffic [15]. This not only places the horse and rider/handler in danger but also endangers surrounding road users or pedestrians. However, changing the behaviour of other road users will be unsuccessful unless we investigate their perceptions of equestrian road users using qualitative and mixed-method approaches. For example, the Australian road safety study identified confusion by vehicle drivers when interpreting equestrian hand signals [15] while a study in South West England found different risk and hazard perceptions between horse riders and drivers not experienced with horses. For example, most drivers had good intensions to pass a horse safely, but when in the middle of doing so started feeling vulnerable themselves and increased their speed [19].

### 4.2. Collisions and Horse Fatalities

It should be kept in mind when discussing the findings of the multivariable logistic regression models that all data compared represent incidents – either near-misses or those directly resulting in injury. We were interested in exploring, firstly, what may increase the odds of an incident becoming a collision and what may increase the odds of an incident leading to a horse sustaining a fatal injury. It is expected that a collision will be more likely to result in injury, however, it has been shown that a significant proportion of horse-related road accidents (44.5%) did not actually involve any physical contact, or impact, between a horse and vehicle [16].

#### 4.2.1. Collisions

Over 20% of incidents in this study involved a collision with another road user although the odds of a collision were seen to reduce over the 10-year period under study. There was a distinct change in odds of a collision over the two time periods, with odds consistently higher between 2010 and 2015 compared to the later years. The BHS Dead Slow campaign was launched in 2016 and may have contributed to a reduction in collisions after this period, due to better driver education [11]. Over the last four years the BHS Safety Team, as part of the Dead Slow campaign, have presented at Road Safety and Advanced Driver Instructor’s national conferences, delivered driver training to large heavy goods vehicle logistic companies and worked with equestrians, equine lawyer specialists and local police forces to hold Road and Rider Awareness evenings. Alternatively, the reduction in collisions could reflect an increase in the frequency of near-misses being reported. Or, perhaps, a combination of both. The evaluation of incidents and incidents that resulted in collisions indicates that incidents do not lead to proportionate collision risk. The South East and Northern Ireland had high odds of collision compared to the North West. While the South East accounted for 15% of incidents, the South West (17%) and West Midlands (14%) did not have significantly higher odds of collisions occurring. Further exploring regional differences in incident reporting and collision odds will help determine areas of the UK where targeted intervention is most needed. Collisions were more likely to occur between late afternoon and early evening (between 15:00 and 19:00) compared to between late morning and early afternoon. This is similar to timings of cycling accidents in Maine, USA (1986–1991) where 44% of accidents occurred between 15:00 and 19:00 and surprisingly few during the morning peak traffic hours [34]. An analysis of road traffic accidents in South Korea (2000–2012) identified that the most dangerous time for travelling was between 16:00 and 18:00 [35]. Additionally, a New Zealand study assessing the impact of the ‘school-run’ on road traffic accidents (1980–2004) found a comparative increase in crash rates during the afternoon but without spatial clustering of crashes around schools [36]. Several factors could contribute to this relationship between collisions and time of day - heavier traffic during the ‘school run’ or peak afternoon commuting time, vehicle drivers may be in more of a rush, be more distracted or fatigued or visibility could be poorer near the end of the day.

Collisions were more likely on roads with speed limits between 20 to 40 mph compared to 60 mph. Additionally, 20% of reporters did not know the speed limit for the road where the incident occurred suggesting that a large proportion of drivers themselves may be unaware of legal speed limits. Even if vehicles were not exceeding the speed limit, they may still have passed the horse unsafely. Passing the horse too closely was the single most contributory factor to a collision, which is in keeping with other UK equestrian road safety research [16]. This, in conjunction with the finding that incidents where vehicle drivers were reported to be exceeding the speed limit had the lowest odds of collisions, suggest that the speed limit of the road itself is not as important as the speed of the vehicle, or arguably their perceived speed by the horse and/or handler and the proximity at which they pass the horse. Interestingly, road rage was associated with lower odds of collisions suggesting that most collisions are unintentional and likely due to a lack of awareness and education rather than intentional harm aimed at the equestrian. However, as indicated by the quotes from several reporters, although not causing physical injury, road rage and near-misses can negatively affect the psychological well-being of both horse and rider. 

Older equestrians were less likely to be involved in collisions than younger equestrians, which was similar to a finding assessing equestrian collisions in Devon County [16]. In the current study, older riders/handlers had significantly greater road experience which may contribute to better hazard perception and more risk-aversive behaviour or potentially better communication with their horse. Lastly, but very importantly, wearing high visibility clothing was associated with considerably lower odds of having a collision. Fluorescent materials in yellow, red and orange are associated with improved detection and recognition of cyclists and pedestrians by drivers during the daytime whereas retroreflective materials, lamps and flashing lights improved detection during the night [37]. Wearing high visibility clothing was associated with a lower likelihood of being involved in a near-miss in a population of equestrians in Devon County [16] and lower rates of accidents in Danish cyclists [38]. However, some studies report conflicting results. A study of cyclists reported no changes in driver behaviour when different clothing, including high visibility jackets, was worn [39]. Behaviour only changed when cyclists were wearing police-type tabards suggesting the journey was being filmed. It may be that certain types of high visibility clothing, with different text messages, may be more effective than others for equestrians. In a UK study of conspicuity equipment worn by horse and rider combinations, it was found that black and white patterned tabards and flashing helmet lights, rather than fluorescent or reflective tabards, significantly increased driver proximity when approaching from the front or rear [40]. Despite this, wearing high visibility, high contrast, retroreflective clothing and/or lights is a simple, yet effective, way in which equestrian road users can make themselves more visible and help contribute to their safety and that of their horses.

#### 4.2.2. Horse Fatalities

While human injury and fatality are clearly of importance, many horse owners perceive their horses as friends, companions, providers of emotional support and as a part of the family [28,41,42]. The experience of losing a horse to euthanasia is distressing and associated with bereavement and grief [43,44,45,46]. Within the DfT police-reported road accident database, horses are classed as vehicles, failing to recognise horses as sentient decision-making animals [15], and horse injury data are not collected. The BHS is the only organisation that collects data on horse injuries resulting from road incidents. In total, over an almost 10-year period, the BHS reports that 892 horses sustained injuries in road incidents; 55% slight, 22% moderate, 8% severe and 15% fatal. However, these incidents are likely only a fraction of those which occur.

The odds of horse fatalities were variable over the 10-year period, with highest odds identified in 2010 and 2016 relative to 2019 but odds generally being higher in the earlier years compared to more recently. In contrast to the collision model, the odds of horse fatality were higher when vehicle drivers were reported to be exceeding the speed limit. This is corroborated by the odds of horse fatality being very strongly associated with collisions. It is well established that pedestrian and cyclist injury severity is worse at greater driver speeds [47,48]. In fact, the risk of death of a pedestrian reaches 10% at 24.1 mph and 50% at 40.6 mph [48]. Additionally, a strong relationship was identified between incidents resulting in horse fatality and rider/ handler injury severity. A fatal injury to a horse is almost 12 times as likely to result in a severe or fatal injury to the rider/handler compared to no injury. Considering that equestrians often use rural roads with speed limits of 60 mph, if this speed limit is being exceeded when a collision occurs then the fatality of the horse, and rider/handler, will be the rule rather than the exception. Horses that fell, whether because of a direct impact with a vehicle or because of slipping or tripping on the road when frightened, were more likely to be injured fatally. Horse falls may be exacerbated by slippery road surfaces where horses are more likely to lose their footing if startled [16]. Interestingly, road rage was once again associated with lower odds of horse fatality.

Finally, loose horses were more likely to be involved in fatal incidents compared to ridden horses or those pulling a horse-drawn vehicle. A loose horse may be a horse that has escaped from their rider/handler (for example if a rider becomes unseated), horses that have broken fencing, those that have not been secured properly or feral free-roaming horses. Feral horse fatalities and injuries are very rarely reported directly to the BHS Horse Incidents website, often because the circumstances surrounding them are not well known. The Dartmoor national park (a 368 square mile area located in South West England) [49] and the New Forest national park (a 220 square mile area located in the South East) [50], both sustain populations of free-roaming native ponies. Pony fatalities and injuries from the national parks are collated independently using reports sent in by the New Forest Verderers (officials in charge of royal forests) or Dartmoor Commoners (farmers and residents with grazing rights on the Moor) and added to the reported horse injury data by the BHS Safety Department for campaigning purposes. In the period between March 2019 and March 2020, 49 incidents involving injury to Dartmoor ponies were reported; 33 (67%) of which were fatal. In a similar timeframe, 38 New Forest ponies were reported injured; 29 (76%) fatally. As a comparison, in 2019 only nine horse fatalities were reported to the BHS Horse Incidents website, three involving ridden horses, two involving horse-drawn vehicles and four involving loose horses. However, all the reported incidents involving loose horses occurred in Wales. It is important to highlight the considerable contribution that free-roaming pony fatalities have on total horse fatalities annually, although these are difficult to assess in context because of missing data or non-reporting.

### 4.3. Limitations

It is important to acknowledge the main limitations of these data. The incident reports are all self-reported incidents and likely represent only a proportion of true accidents and near-misses. There is also a likely bias in reporting, with more serious incidents also more likely to be reported or individuals’ awareness of the incident reporting website and their personal motivation influencing whether an incident is reported. Additionally, many observations are subjective, specifically when relating to vehicle speed, passing distance and injury severity. For example, there is evidence that pedestrians crossing the road tend to overestimate the speed of a single vehicle travelling between 12 and 30 mph, but underestimate speeds faster than 30 mph [51]. However, when more than one vehicle is involved, underestimation is more likely at lower vehicle speeds. Finally, the context in which some of the questions were asked was not ideal. However, this incident reporting system does provide valuable information about the challenges that equestrians face when using roads. In particular, the circumstances which have led to physical injury or mental distress to the rider/handler and horse and the scope to promote simple and cost-effective interventions, such as high-visibility clothing, which could reduce the frequency of horse and human injury when interacting with other road users.

## 5. Conclusions

A considerable proportion of equestrians reported road rage by other road users, vehicles passing at close proximity and exceeding the speed limit to be present in horse-related road incidents. Close passing distances were associated with higher odds of a collision while excess speeds were associated with higher odds of horse fatality. Although the odds of collision-related incidents have reduced over time, driver awareness of how to pass horses safely on the roads in the UK is still lacking. Identifying ways in which to reach the wider vehicle-driving community, helping to change their behaviour around horses, and securing funding for such projects is necessary. The use of conspicuous high visibility clothing was associated with lower collision odds and is a simple step that all equestrians can take to make themselves and their horses more visible. Loose horses contribute significantly to road-related horse fatalities; however, free-roaming/feral horse incidents are severely under-represented and run the risk of exclusion from any future road law and policy changes. Horse fatalities and rider/handler injury are intricately linked and reducing the risk of injury to horses will serve to reduce human injury and loss of life.

## Figures and Tables

**Table 1:** A summary of equestrian-related road incident data reported to the British Horse Society between 2010 and 2020.

Variable	Number of Incidents	Percentage of Incidents	95% Confidence Interval (%)
Incident Details			
**Incident month (*n* = 4093)**			
January	281	6.9	6.1, 7.6
February	303	7.4	6.6, 8.2
March	408	10.0	9.1, 10.9
April	339	8.3	7.4, 9.1
May	395	9.7	8.7, 10.6
June	362	8.8	8.0, 9.7
July	411	10.0	9.1, 11.0
August	415	10.1	9.2, 11.1
September	289	7.1	6.3, 7.8
October	332	8.1	7.3, 8.9
November	341	8.3	7.5, 9.2
December	217	5.3	4.6, 6.0
**Incident season (*n* = 4093)**			
Winter (Dec–Feb)	801	19.6	18.4, 20.8
Spring (Mar–May)	1142	27.9	26.5, 29.3
Summer (Jun–Aug)	1188	29.0	27.6, 30.4
Autumn (Sep–Nov)	962	23.5	22.2, 24.4
**Incident year (*n* = 4107)**			
2010	88	2.1	1.7, 2.6
2011	233	5.7	5.0, 6.4
2012	180	4.4	3.8, 5.0
2013	214	5.2	4.5, 5.9
2014	299	7.3	6.5, 8.1
2015	309	7.5	6.7, 8.3
2016	372	9.1	8.2, 9.9
2017	375	9.1	8.2, 10.0
2018	688	16.8	15.6, 17.9
2019	916	22.3	21.0, 23.6
2020	433	10.5	9.6, 11.5
**Incident year category (*n* = 4107)**			
2010–2015	1323	32.2	30.8, 33.6
2016–2020	2784	67.8	66.4, 69.2
**Time of incident (*n* = 3987)**			
20:00–05:00	48	1.2	0.9, 1.5
06:00–09:00	483	12.1	11.1, 13.1
10:00–14:00	2187	54.9	53.3, 56.4
15:00–19:00	1269	31.8	30.4, 33.3
**Incident region (*n* = 4107)**			
South West	691	16.8	15.7, 18.0
South East	620	15.1	14.0, 16.2
West Midlands	574	14.0	12.9, 15.0
East	439	10.7	9.7, 11.6
Yorkshire & Humber	440	10.7	9.8, 11.7
North West	418	10.2	9.3, 11.1
Wales	292	7.1	6.3, 7.9
Scotland	276	6.7	6.0, 7.5
East Midlands	171	4.2	3.6, 4.8
North East	115	2.8	2.3, 3.3
Northern Ireland	29	0.7	0.5, 1.0
Isle of Man	26	0.6	0.4, 0.9
London	16	0.4	0.2, 0.6
**Incident reported to police? (*n* = 4029)**			
No	2321	57.6	56.1, 59.1
Yes	1708	42.4	40.9, 43.9
**Incident road type (*n* = 4045)**			
Minor	1956	48.4	46.8, 49.9
Secondary	813	20.1	18.9, 21.3
Main	757	18.7	17.5, 19.9
Trunk	34	0.8	0.6, 1.1
Other	217	5.4	4.7, 6.1
Unknown	268	6.6	5.9, 7.4
**Incident area type (*n* = 3984)**			
Rural	2963	74.4	73.0, 75.7
Urban	209	5.2	4.6, 5.9
Suburban	635	15.9	14.8, 17.1
Wooded	93	2.3	1.9, 2.8
Other	84	2.1	1.7, 2.6
**Road speed limit in miles per hour (*n* = 4107)**			
20	156	3.8	3.2, 4.4
30	1157	28.2	26.8, 29.5
40	380	9.3	8.4, 10.1
50	201	4.9	4.2, 5.6
60	1331	32.4	31.0, 33.8
70	41	1.0	0.7, 1.3
Unknown	841	20.5	19.2, 21.7
**Weather condition (*n* = 3894)**			
Bright	660	16.9	15.8, 18.1
Dry	2864	73.5	72.2, 74.9
Fog/Ice/Snow	30	0.8	0.5, 1.0
Wet	340	8.7	7.8, 9.6
**Road surface condition (*n* = 3797)**			
Damaged	116	3.1	2.5, 3.6
New	942	24.8	23.4, 26.2
Worn	2739	72.1	70.7, 73.6
**Visibility condition (*n* = 3854)**			
Good	3503	90.9	90.0, 91.8
Fair	294	7.6	6.8, 8.5
Poor	57	1.5	1.1, 1.9
**Incident included road rage (*n* = 3916)**			
No	2337	59.7	58.1, 61.2
Yes	1579	40.3	38.8, 41.9
			
**Did driver lose control of vehicle? (*n* = 3855)**			
No	3495	90.7	89.7, 91.6
Yes	360	9.3	8.4, 10.3
**Was vehicle driver exceeding the speed limit? (*n* = 3843)**			
No	2302	59.9	58.4, 61.5
Yes	1541	40.1	38.5, 41.6
**Did the vehicle driver pass too close? (*n* = 3951)**			
No	626	15.8	14.7, 17.0
Yes	3325	84.2	83.0, 85.3
**Vehicle speed and passing distance (*n* = 3801)**			
Speeding & passing too close	1323	34.8	33.3, 36.3
Speeding only	192	5.1	4.4, 5.7
Passing too close only	1860	48.9	47.3, 50.5
Neither speeding nor passing too close	426	11.2	10.2, 12.2
**Did the rider/handler lose control of the horse? (*n* = 3829)**			
No	2559	66.8	65.3, 68.3
Yes	1270	33.2	31.7, 34.7
**Details of the main horse involved in the incident**			
**Horse age category in quartiles (*n* = 3853)**			
up to 7 years	979	25.4	24.0, 26.8
8–11 years	1122	29.1	27.7, 30.6
12–15 years	907	23.5	22.2, 24.9
>15 years	845	21.9	20.6, 23.2
**Frequency of road use (*n* = 3883)**			
More than once/week	2846	73.3	71.9, 74.7
Weekly	837	21.6	20.3, 22.8
Monthly	98	2.5	2.0, 3.0
Other	102	2.6	2.1, 3.1
**Horse use (*n* = 4096)**			
Ridden	3681	89.9	88.9, 90.8
Horse-drawn vehicle	117	2.9	2.3, 3.4
Led by person on foot	251	6.1	5.4, 6.9
Loose (absence of human handler)	47	1.1	0.8, 1.5
**Did horse fall? (*n* = 4014)**			
No	3661	91.2	90.3, 92.1
Yes	353	8.8	7.9, 9.7
**Severity of injury to horse (*n* = 4089)**			
None	3197	78.2	76.9, 79.5
Mild	494	12.1	11.1, 13.1
Moderate	194	4.7	4.1, 5.4
Severe	74	1.8	1.4, 2.2
Fatal	130	3.2	2.6, 3.7
**Did the horse receive veterinary treatment? (*n* = 4044)**			
No	3696	91.4	90.5, 92.5
Yes	348	8.6	7.7, 9.5
**Collision between the horse, rider/handler or horse-drawn vehicle and driver of vehicle (*n* = 4094)**			
No	3239	79.1	77.9, 80.4
Yes	855	20.9	19.6, 22.1
**Area of the horse was struck in the collision (*n* = 771)**			
Front	62	8.0	6.1, 10.0
Rear	486	63.0	59.6, 66.4
Side	223	28.9	25.7, 32.1
**Details of the main rider/handler involved in the incident**			
**Rider/handler experience on the road (*n* = 3814)**			
0–5 years	206	5.4	4.7, 6.1
6–10 years	370	9.7	8.8, 10.6
11–15 years	457	12.0	11.0, 13.0
15 years+	2781	72.9	71.5, 74.3
**Rider/handler passed BHS Riding and Road safety test? (*n* = 3684)**			
No	2375	64.5	62.9, 66.0
Yes	1309	35.5	34.0, 37.1
**Rider/handler age category in quartiles (*n* = 3846)**			
up to 27 years	965	25.1	23.7, 26.5
28–40 years	1012	26.3	24.9, 27.7
41–50 years	992	25.8	24.4, 27.2
>50 years	877	22.8	21.5, 24.1
**Rider/handler age category (*n* = 3846)**			
up to 15 years	134	3.5	2.9, 4.1
16–25 years	692	8.0	16.8, 19.2
26–35 years	720	18.7	17.5, 20.0
36–45 years	952	24.8	23.4, 26.1
46–55 years	819	21.3	20.0, 22.6
56–65 years	441	11.5	10.5, 12.5
>65 years	88	2.3	1.8, 2.8
**Rider/handler gender (*n* = 4107)**			
Female	3646	88.8	87.8, 89.7
Male	222	5.4	4.7, 6.1
Unknown	239	5.8	5.1, 6.5
**Did the rider/handler fall (*n* = 4093)**			
No	3409	83.3	82.1, 84.4
Yes	684	16.7	15.6, 17.9
**Severity of injury to rider/handler (*n* = 4084)**			
None	3174	77.7	76.4, 79.0
Mild	447	10.9	10.0, 11.9
Moderate	320	7.8	7.0, 8.7
Severe	122	3.0	2.5, 3.5
Fatal	21	0.5	0.3, 0.8
**Type of medical treatment received by rider/handler (*n* = 4040)**			
None	3568	88.3	87.3, 89.3
A&E	158	3.9	3.3, 4.5
Road Ambulance	161	4.0	3.4, 4.6
Air Ambulance	43	1.1	0.7, 1.4
GP	110	2.7	2.2, 3.2
**High visibility clothing worn by the rider/handler or horse? (*n* = 3958)**			
No	350	8.8	8.0, 9.7
Yes	3608	91.2	90.3, 92.0
**Riding helmet worn by rider/handler? (*n* = 3898)**			
No	184	4.7	4.1, 5.4
Yes	3714	95.3	94.6, 95.9
**Body protector worn by rider/handler? (*n* = 3777)**			
No	3685	97.7	97.2, 98.1
Yes	88	2.3	1.9, 2.8
**Details of the person reporting the incident**			
**Reporter’s involvment in incident (*n* = 4018)**			
Friend	74	1.8	1.4, 2.3
Motorist	139	3.5	2.9, 4.0
Police	35	0.9	0.6, 1.2
Rider/handler	3472	86.4	85.4, 87.5
Witness	142	3.5	3.0, 4.1
Other	156	3.9	3.3, 4.5
**Does reporter have liability insurance? (*n* = 3796)**			
No	305	8.0	7.2, 8.9
Yes	3491	92.0	91.1, 92.8
**Is reporter a British Horse Society member? (*n* = 4106)**			
No	2665	64.9	63.4, 66.4
Yes	1441	35.1	33.6, 36.6

**Table 2:** Multivariable mixed-effects logistic regression modelling, including reporter as a random effect, of the incident- and rider/handler-related variables associated with higher odds of vehicle collisions in road incidents (*n* = 3463) reported to the British Horse Society between 2010 and 2020.

Variable	Coefficient	Standard Error	Odds Ratio (OR)	95% Confidence Interval (OR)	Wald *p*-Value; LRS *p*-Value ^1^
**Incident year category**					
2010–2015	1.3	0.2	3.6	2.5, 5.2	**<0.001**
2016–2020	Reference				
**Time of incident**					**0.04**
20:00–05:00	−0.97	0.96	0.4	0.1, 2.5	0.314
06:00–09:00	0.3	0.2	1.3	0.8, 2.1	0.217
10:00–14:00	Reference				
15:00–19:00	0.4	0.2	1.4	1.1, 2.0	0.016
**Incident region**					**0.04**
North West	Reference				
South West	0.6	0.3	1.8	1.0, 3.4	0.056
South East	0.8	0.3	2.1	1.2, 3.9	0.016
West Midlands	0.5	0.3	1.7	0.9, 3.2	0.085
East	0.4	0.3	1.5	0.8, 3.0	0.197
Yorkshire & Humber	0.4	0.3	1.5	0.8, 2.8	0.247
Wales	0.4	0.4	1.4	0.7, 3.1	0.344
Scotland	−0.003	0.4	1.0	0.5, 2.1	0.994
East Midlands	0.08	0.4	1.1	0.5, 2.6	0.856
North East	0.7	0.5	1.1	0.4, 2.8	0.881
Northern Ireland	2.5	0.8	11.7	2.4, 56.8	0.002
London	1.9	1.2	6.9	0.6, 78.3	0.120
**Road speed limit in miles per hour**					**0.002**
20	1.2	0.4	3.2	1.5, 6.8	0.002
30	0.6	0.2	1.9	1.3, 2.8	0.001
40	0.7	0.3	2.0	1.2, 3.3	0.012
50	0.4	0.4	1.6	0.8, 3.1	0.215
60	Reference				
70	0.8	0.7	2.3	0.5, 9.6	0.257
Unknown	0.7	0.2	2.1	1.4, 3.2	0.001
**Incident included road rage**					
No	Reference				
Yes	−1.4	0.2	0.2	0.2, 0.4	**<0.001**
**Vehicle speed and passing distance**					**<0.001**
Speeding only	Reference				
Speeding & passing too close	1.5	0.5	4.4	1.7, 11.7	0.003
Passing too close only	2.9	0.5	18.3	6.5, 51.6	<0.001
Neither speeding nor passing too close	1.9	0.5	6.9	2.4, 20.0	<0.001
**Rider/handler age in years (continuous)**	−0.02	0.006	0.97	0.96, 0.99	**<0.001**
**High visibility clothing worn by the rider/handler or horse**					
No	Reference				
Yes	−1.4	0.3	0.2	0.1, 0.4	**<0.001**

^1^ LRS—Likelihood ratio statistic. *p*-Values in bold represent LRS-*p* <0.05.

**Table 3:** Multivariable logistic regression modelling of incident-, horse- and rider/handler-related variables associated with higher odds of horse fatality road incidents (*n* = 3709) reported to the British Horse Society between 2010 and 2020.

Variable	Coefficient	Standard Error	Odds Ratio (OR)	95% Confidence Interval (OR)	Wald *p*-Value; LRS *p*-value ^1^
**Incident year**					**0.002**
2010	3.0	0.9	19.8	3.3, 118.3	0.001
2011	2.4	0.9	10.7	1.9, 61.1	0.008
2012	2.3	0.9	9.9	1.8, 54.3	0.008
2013	2.4	0.9	10.8	1.8, 62.8	0.008
2014	1.0	0.9	2.7	0.4, 17.3	0.281
2015	2.1	0.9	8.1	1.4, 48.3	0.022
2016	2.5	0.9	11.8	2.2, 63.2	0.004
2017	1.1	1.0	2.9	0.4, 22.2	0.301
2018	0.8	1.0	2.3	0.3, 16.0	0.400
2019	Reference				
2020	1.5	1.0	4.4	0.6, 33.4	0.147
**Incident included road rage**					
No	Reference				
Yes	−1.6	0.6	0.2	0.1, 0.6	**0.005**
**Was vehicle driver exceeding the speed limit?**					
No	Reference				
Yes	0.9	0.3	2.3	1.2, 4.6	**0.013**
**Collision between the horse, rider/handler or horse-drawn vehicle and driver of vehicle**					
No	Reference				
Yes	4.3	0.7	73.2	17.2, 310.9	**<0.001**
**Did the horse fall?**					
No	Reference				
Yes	1.6	0.3	5.2	2.6, 10.1	**<0.001**
**Horse use**					**<0.001**
Ridden/Horse-drawn vehicle	Reference				
Led by person on foot	0.4	0.7	1.5	0.4, 5.7	0.595
Loose (absence of human handler)	4.3	0.7	75.0	19.8, 284.0	<0.001
**Severity of injury to rider/handler**					**<0.001**
None	Reference				
Mild	0.7	0.5	2.0	0.8, 5.2	0.142
Moderate	1.5	0.5	4.3	1.7, 11.0	0.002
Severe to fatal	2.5	0.5	11.8	4.1, 34.0	<0.001

^1^ LRS—Likelihood ratio statistic. *p*-Values in bold represent LRS-*p* <0.05.

## Data Availability

The data and code that support the findings of this study are openly available in [BHS-equestrianroadsafety/horse-collisions-injuries] at http://doi.org/10.5281/zenodo.4286186.

## Supplementary Materials

The following are available online at https://www.mdpi.com/2076-2615/10/12/2403/s1. Form S1. The British Horse Society Road Incident Form. Table S2. Detailed outputs of significant clusters from the space-time permutation model. Detail includes the location and time period in which the cluster occurred and includes aggregated area and period calculations. Table S3. Univariable mixed-effects logistic regression modelling, including reporter as a random effect, of incident-, horse- and rider/handler-related variables associated with higher odds of vehicle collisions in incidents reported to the British Horse Society between 2010 and 2020. Table S4. Univariable logistic regression modelling of incident-, horse- and rider/handler-related variables associated with higher odds of horse fatality road incidents reported to the British Horse Society between 2010 and 2020.
