# Supplementary material for: Equestrian Road Safety in the United Kingdom: Factors Associated with Collisions and Horse Fatalities
Source: Animals (Basel). 2020 Dec 15;10(12):2403. doi: 10.3390/ani10122403 (PMC7765430; doi:10.3390/ani10122403)
Supplement: Supplementary file 1 [file animals-10-02403-s001.pdf]

Supplementary information

Form S1 The British Horse Society Road Incident Form

Reporting of Equestrian Incidents
Road Incident Form

The British Horse Society

Please fill in the form below circling the most suitable answer when required

* - Denotes a required field – this information **MUST** be included to enable the incident to be added to our website and database.

Incident Details

Date and Time of Incident *	
County *	
Specific Location of incident<br>(Please include nearest post code) *	
Was the incident reported to the police?	Y / N
Police incident report number (if applicable)	
Is any further police action being taken?	Y / N
Do you have public liability insurance?	Y / N

Personal Details

Forename	
Surname	
BHS Membership Number	
Address	
Telephone Number	
Email Address	
Involvement with incident	Rider / Motorist / Witness / Friend / Police / Other
If 'Other' please give details	

Circumstances

Type of Road	Trunk / Main / Secondary / Minor / Unknown / Other
Type of Area	Urban / Suburban / Rural / Wooded / Other
Speed limit where incident happened	20mph / 30mph / 40mph / 50mph / 60mph / 70mph / unknown
Weather Conditions	Dry / Wet / Icy / Snowy / Fog / Bright
Road Surface conditions	New / Worn / Damaged
Visibility Conditions	Good / Fair / Poor
Drivers Road Rage	Y / N
Loss of control of vehicle	Y / N
Vehicle exceeding speed limit	Y / N
Vehicle passing too close to horse and rider	Y / N
Loss of control of horse	Y / N
Please give further details of the incident here:	

Horses
(Please complete the following for the main horse involved. There will be space below to detail any other horses present)

Number of horses involved	
Age of main horse involved	
Frequency of main horse involved ridden on roads	More than once a week / weekly / monthly / other
Horse	Ridden / driven / leading / loose
Horse fall	Y / N
Severity of horse’s injury	None / mild / moderate / severe / fatal
Veterinary assistance required	Y / N
Veterinary practice contact information	
Collision with vehicle	Y / N
**Area of horse struck by vehicle**	Front / side / rear

Humans

(Please complete the following for the main rider / handler involved. There will be space below to detail any other humans present)

Number of people involved	
Experience of rider / handler on the road	0-5years / 5-10years / 10 -15years / 15 years +
Had rider / handler passed BHS Riding and Road safety Test?	Y / N
Age of main rider / handler	
Gender	
Rider fall	Y / N
Severity of rider / handler injury	None / Mild / Moderate / Severe / Fatal
Medical help sought	None / GP / Hospital / Ambulance / Air Ambulance

Details of other parties involved. Where possible use the questions above as a guide

Safety Equipment

(Please complete for the main handler/ horse partnership involved. There will be space below to detail any other partnerships present

Hi – viz	Y / N
Type of hi – viz worn	
Age of hi - viz	
Hat	Y / N
Was the hat displaced	Y / N
Hat type	PAS015 / BSEN1384 / ASTMF1163 / Other

Age of hat	
Body protector worn?	Y / N
Type of body protector	BETA level 1 / BETA level 2 / BETA level 3 / BSEN13158
Age of body protector	
Items of tack worn e.g. saddle/bridle	
Type of tack worn	Leather / synthetic / other
Approximate age of tack	

Please detail any safety equipment worn by other partnerships involved

Any Other Information

Please give any other relevant information?

Data Protection

Are you happy to receive occasional emails from the BHS about its work?	Y / N
Are you happy to receive emails from other organisations that are supportive of the BHS?	Y / N

The BHS will retain your details on a database. The BHS and its subsidiaries may like to contact you with information about our charitable activities and how you could help us. Are you happy for your details to be used in this way?	Y / N
Are you happy for the BHS to share your information with other organisations that are supportive of the BHS?	Y / N
We will share statistical and incident data we collect with other appropriate governing bodies and organisations. This data will be anonymous: your name, home address or other identifying information, or the details of anyone else that you have given us, will not be passed on to anyone outside the BHS without your consent	
Are you happy to let us pass on your identifying details on to other organisation, carefully selected by the BHS for the purposes of improving equestrian safety	Y / N

Signature ……………………………………………………………………….

Date ………………………………………………………………………

Table S2. Detailed outputs of significant clusters from the space-time permutation model. Detail includes the location and time period in which the cluster occurred and includes aggregated area and period calculations.

Clusterid	Centroid	Radius (km)	Timeframe start	Timeframe end	Time span (days)	Incidents observed	Incidents expected	P-value
1	50.609306 N, 3.487826 W	13.35	03 March 2018	02 April 2019	395	116	24.91	<0.001
2	53.837829 N, 1.710986 W	4.23	03 August 2016	02 June 2017	303	35	4.47	<0.001
3	51.685382 N, 0.150335 E	36.63	03 December 2010	02 July 2013	942	58	20.63	<0.001
4	51.592563 N, 0.702228 W	4.92	03 October 2018	02 January 2019	91	13	1.12	<0.001
5	52.613346 N, 0.232983 E	9.9	03 March 2015	02 May 2016	426	13	1.2	<0.001
6	58.204757 N, 6.281633 W	0.27	03 October 2019	02 November 2019	30	7	0.18	<0.001
7	51.445423 N, 0.767927 W	11.34	03 December 2012	02 January 2013	30	6	0.13	<0.01
8	54.134967 N, 2.655514 W	4.68	03 August 2016	02 September 2016	30	5	0.062	<0.01
9	51.756583 N, 2.485708 W	0.59	03 September 2019	02 October 2019	29	6	0.14	<0.01
10	53.311592 N, 2.677969 W	23.71	03 September 2019	02 June 2020	273	35	11.741	<0.05
11	53.800219 N, 2.824627 W	10.39	03 August 2020	02 September 2020	30	7	0.29	<0.05
	**Mean**	11 km		**Mean**	234 days			
	**Median**	9.9 km		**Median**	91 days			
	**Range**	0.27 - 36.63 km		**Range**	29 - 942 days			

Table S3. Univariable mixed-effects logistic regression modelling, including reporter as a random effect, of incident-, horse- and rider/handler-related variables associated with higher odds of vehicle collisions in incidents reported to the British Horse Society between 2010 and 2020.

Variable	Coefficient	Standard error	Odds ratio (OR)	95% confidence interval (OR)	Wald P-value
**Incident details**					
**Incident month**					**0.057**
January	0.8	0.4	2.2	1.05, 4.4	0.037
February	0.9	0.4	2.4	1.2, 4.9	0.016
March	0.3	0.3	1.3	0.7, 2.5	0.448
April	0.7	0.4	2.1	1.03, 4.1	0.04
May	0.2	0.3	1.3	0.6, 2.5	0.494
June	0.1	0.3	1.2	0.6, 2.3	0.669
July	Reference				
August	0.7	0.3	1.9	1.01, 3.7	0.048
September	1.1	0.4	3.1	1.5, 6.3	0.002
October	0.7	0.4	2.0	1.0, 3.9	0.06
November	0.6	0.4	1.7	0.9, 3.5	0.113
December	1.0	0.4	2.8	1.3, 6.1	0.007
**Incident season**					**0.012**
Winter (Dec – Feb)	0.6	0.2	1.8	1.2, 2.8	0.005
Spring (Mar – May)	0.1	0.2	1.1	0.8, 1.7	0.553
Summer (Jun – Aug)	Reference				
Autumn (Sep – Nov)	0.5	0.2	1.6	1.1, 2.4	0.017
**Incident year**					**<0.001**
2010	3.6	0.5	37.0	13.4, 102.0	<0.001
2011	2.0	0.3	7.5	3.8, 14.5	<0.001
2012	2.7	0.4	14.3	6.7, 30.4	<0.001
2013	1.9	0.3	6.8	3.5, 13.5	<0.001
2014	2.0	0.3	7.3	3.9, 13.5	<0.001
2015	1.2	0.3	3.3	1.8, 5.9	<0.001
2016	0.8	0.3	2.3	1.3, 3.9	0.003
2017	0.6	0.3	1.9	1.1, 3.3	0.03
2018	0.4	0.2	1.4	0.9, 2.3	0.143
2019	Reference				
2020	-0.2	0.3	0.9	0.5, 1.5	0.589
**Incident year category**					
2010-2015	1.8	0.2	5.9	4.0, 8.7	**<0.001**
2016-2020	Reference				
**Time of incident**					**0.022**
>7pm-5am	1.0	0.6	2.7	0.8, 8.4	0.096
>5am-9am	0.4	0.2	1.5	1.0, 2.4	0.052
>9am-2pm	Reference				
>2pm-7pm	0.4	0.2	1.5	1.1, 2.0	0.012

**Incident region**					**0.007**
South West	0.9	0.3	2.5	1.3, 4.7	0.004
South East	1.1	0.3	2.9	1.5, 5.4	0.001
West Midlands	0.7	0.3	1.9	1.02, 3.6	0.043
East	1.3	0.3	3.6	1.9, 7.1	<0.001
Yorkshire & Humber	0.8	0.3	2.2	1.1, 4.3	0.022
North West	Reference				
Wales	1.1	0.4	2.9	1.4, 6.2	0.006
Scotland	0.5	0.4	1.7	0.8, 3.7	0.152
East Midlands	0.4	0.4	1.5	0.6, 3.7	0.341
North East	0.8	0.5	2.2	0.8, 5.8	0.114
Northern Ireland	1.9	0.8	6.4	1.2, 33.5	0.028
Isle of Man	-2.3	1.6	0.1	0.005, 2.4	0.155
London	3.3	1.1	27.2	3.2, 229.6	0.002
**Incident road type**					**0.433**
Minor	-0.1	0.2	0.9	0.6, 1.4	0.802
Secondary	-0.3	0.3	0.7	0.4, 1.2	0.180
Main	Reference				
Trunk	1.0	1.0	2.8	0.4, 21.7	0.318
Other	-0.2	0.4	0.8	0.4, 1.6	0.549
Unknown	-0.5	0.3	0.6	0.3, 1.3	0.193
**Incident area type**					**0.542**
Rural	0.1	0.2	1.1	0.7, 1.6	0.712
Urban	0.2	0.4	1.2	0.6, 2.5	0.574
Suburban	Reference				
Wooded	0.7	0.5	2.1	0.8, 5.1	0.115
Other	0.4	0.5	1.5	0.6, 4.0	0.424
**Road speed limit in miles per hour**					**0.01**
20	0.7	0.4	2.1	1.01, 4.4	0.048
30	0.3	0.2	1.3	0.9, 1.9	0.129
40	0.4	0.3	1.6	0.9, 2.6	0.091
50	0.4	0.3	1.6	0.8, 3.0	0.197
60	Reference				
70	1.0	0.7	2.7	0.7, 10.3	0.142
Unknown	0.8	0.2	2.2	1.5, 3.3	<0.001
**Weather condition**					**0.042**
Bright	0.5	0.2	1.6	1.1, 2.3	1.1, 2.3
Dry	Reference				
Fog/Ice/Snow	0.1	0.8	1.1	0.2, 5.6	0.2, 5.6
Wet	-0.3	0.3	0.8	0.5, 1.3	0.5, 1.3
**Road surface condition**					**0.117**
Damaged	-0.4	0.4	0.7	0.3, 1.6	0.35
New	0.3	0.2	1.3	1.0, 1.8	0.08
Worn	Reference				

**Visibility condition**					**0.186**
Good	Reference				
Fair	-0.1	0.3	0.9	0.5, 1.5	0.696
Poor	0.9	0.5	2.5	0.9, 6.9	0.076
**Incident included road rage**					
No	Reference				
Yes	-1.8	0.2	0.2	0.1, 0.3	**<0.001**
**Did driver lose control of vehicle?**					
No	Reference				
Yes	0.005	0.3	1.0	0.6, 1.7	**0.985**
**Was vehicle driver exceeding the speed limit?**					
No	Reference				
Yes	-1.3	0.2	0.3	0.2, 0.4	**<0.001**
**Did the vehicle driver pass too close?**					
No	Reference				
Yes	0.3	0.2	1.4	0.9, 2.1	**0.097**
**Vehicle speed and passing distance**					**<0.001**
Speeding & passing too close	1.1	0.5	3.0	1.2, 7.5	0.021
Speeding only	Reference				
Passing too close only	2.3	0.5	10.3	4.0, 26.6	<0.001
Neither speeding nor passing too close	2.1	0.5	7.8	2.8, 21.5	<0.001
**Did the rider/handler lose control of the horse?**					
No	Reference				
Yes	-0.03	0.2	1.0	0.7, 1.4	**0.855**
**Details of the main horse involved in the incident**					
**Horse age in years (continuous)**	-0.04	0.01	0.96	0.94, 0.99	**0.009**
**Horse age category in quartiles**					**0.136**
up to 7 years	0.5	0.2	1.6	1.04, 2.4	0.029
8-11 years	0.4	0.2	1.5	1.0, 2.2	0.065
12-15 years	0.2	0.2	1.2	0.8, 1.9	0.359
>15 years	Reference				
**Frequency of road use**					**0.176**
More than once/week	Reference				
Weekly	-0.2	0.2	0.8	0.6, 1.2	0.26
Monthly	0.3	0.4	1.3	0.5, 3.1	0.549
Other	0.7	0.4	2.0	0.9, 4.6	0.088

**Horse use**					**<0.001**
Ridden	Reference				
Horse-drawn vehicle	1.1	0.4	3.1	1.3, 7.3	0.009
Leading	-0.3	0.3	0.7	0.4, 1.4	0.345
Loose	7.5	1.2	1732.6	174.0, 17250.1	<0.001
**Details of the main rider/handler involved in the incident**					
**Rider/handler experience on the road**					**0.034**
0-5 years	0.7	0.3	2.1	1.1, 3.9	0.027
6-10 years	0.3	0.3	1.3	0.8, 2.2	0.251
11-15 years	0.4	0.2	1.6	1.05, 2.5	0.031
15 years+	Reference				
**Rider/handler age in years (continuous)**	-0.02	0.005	0.98	0.97, 0.99	**<0.001**
**Rider/handler age category in quartiles**					**0.0002**
up to 27 years	0.9	0.2	2.4	1.6, 3.7	<0.001
28-40 years	0.3	0.2	1.3	0.9, 1.9	0.231
41-50 years	0.1	0.2	1.1	0.7, 1.8	0.541
>50 years	Reference				
**Rider/handler gender**					**<0.001**
Female	Reference				
Male	1.4	0.3	4.0	2.1, 7.6	<0.001
Unknown	1.6	0.3	4.9	2.6, 9.1	<0.001
**High visibility clothing worn by the rider/handler or horse?**					
No	Reference				
Yes	-2.3	0.3	0.1	0.06, 0.17	**<0.001**

Table S4. Univariable logistic regression modelling of incident-, horse- and rider/handler-related variables associated with higher odds of horse fatality road incidents reported to the British Horse Society between 2010 and 2020.

Variable	Coefficient	Standard error	Odds ratio (OR)	95% confidence interval (OR)	Wald P-value
**Incident details**					
**Incident month**					**0.001**
January	2.0	0.6	7.2	2.1, 24.9	0.002
February	1.4	0.7	4.2	1.2, 15.3	0.028
March	1.5	0.6	4.6	1.3, 15.9	0.016
April	Reference				
May	0.5	0.7	1.7	0.4, 7.0	0.442
June	0.6	0.7	1.9	0.5, 7.6	0.372
July	1.4	0.6	4.3	1.2, 14.8	0.023
August	0.8	0.7	2.2	0.6, 8.4	0.245
September	1.7	0.6	5.7	1.6, 20.1	0.007
October	1.0	0.7	2.8	0.8, 10.5	0.137
November	1.5	0.6	4.4	1.3, 15.7	0.021
December	1.9	0.7	6.6	1.8, 23.6	0.004
**Incident season**					**0.003**
Winter (Dec – Feb)	0.9	0.3	2.4	1.4, 3.9	0.001
Spring (Mar – May)	Reference				
Summer (Jun – Aug)	0.1	0.3	1.1	0.7, 1.9	0.687
Autumn (Sep – Nov)	0.5	0.3	1.7	1.0, 2.8	0.05
**Incident year**					**<0.001**
2010	3.1	0.4	21.9	9.2, 51.8	<0.001
2011	2.1	0.4	8.6	3.8, 19.4	<0.001
2012	2.3	0.4	10.1	4.4, 23.3	<0.001
2013	1.7	0.5	5.4	2.2, 13.3	<0.001
2014	1.3	0.5	3.5	1.4, 8.7	0.007
2015	1.2	0.5	3.4	1.4, 8.4	0.009
2016	1.6	0.4	4.8	2.1, 10.9	<0.001
2017	0.6	0.5	1.9	0.7, 5.2	0.201
2018	0.5	0.5	1.6	0.7, 4.0	0.275
2019	Reference				
2020	0.3	0.5	1.4	0.5, 4.0	0.514
**Incident year category**					
2010-2015	1.3	0.2	3.6	2.5, 5.1	**<0.001**
2016-2020	Reference				
**Time of incident**					**<0.001**
>7pm-5am	2.8	0.4	16.8	8.0, 35.4	<0.001
>5am-9am	0.7	0.3	2.1	1.2, 3.7	0.014
>9am-2pm	Reference				
>2pm-7pm	0.7	0.2	2.0	1.3, 3.1	0.002

**Incident region**					**0.054**
South West	0.3	0.4	1.3	0.6, 2.9	0.539
South East	0.7	0.4	2.1	1.0, 4.5	0.058
West Midlands	0.1	0.4	1.1	0.5, 2.6	0.803
East	0.6	0.4	1.8	0.8, 4.2	0.148
Yorkshire & Humber	Reference				
North West	0.3	0.5	1.3	0.5, 3.2	0.571
Wales	1.1	0.4	3.0	1.3, 6.8	0.009
Scotland	0.5	0.5	1.6	0.6, 4.1	0.316
East Midlands	0.1	0.6	1.1	0.3, 3.8	0.821
North East	0.2	0.7	1.3	0.3, 4.8	0.712
Northern Ireland	0.5	1.1	1.7	0.2, 14.0	0.617
London	2.4	0.7	11.1	2.7, 45.6	0.001
**Incident road type**					**<0.001**
Minor	Reference				
Secondary	0.7	0.2	2.0	1.3, 3.1	0.003
Main	0.7	0.2	2.0	1.3, 3.2	0.003
Other	-0.2	0.5	0.9	0.3, 2.4	0.762
Unknown	-1.8	1.0	0.2	0.02, 1.2	0.081
**Incident area type**					**0.416**
Rural	-0.4	0.4	0.7	0.3, 1.4	0.294
Urban	Reference				
Suburban	-0.2	0.4	0.8	0.4, 1.9	0.646
Wooded	-0.6	0.8	0.6	0.1, 2.7	0.467
Other	0.5	0.6	1.6	0.5, 5.0	0.428
**Road speed limit in miles per hour**					**<0.001**
20	1.2	1.0	3.4	0.5, 25.3	0.231
30	2.0	1.0	7.2	1.0, 54.7	0.056
40	1.7	1.1	5.6	0.7, 45.6	0.11
50	1.2	1.0	3.4	0.5, 25.4	0.227
60	Reference				
70	2.5	1.2	12.2	1.2, 120.2	0.033
Unknown	2.3	1.0	9.5	1.3, 69.3	0.027
**Weather condition**					**0.028**
Bright	0.6	0.2	1.8	1.1, 2.9	0.02
Dry	Reference				
Fog/Ice/Snow	1.6	0.6	5.2	1.5, 17.6	0.008
Wet	0.1	0.4	1.1	0.5, 2.4	0.752
**Visibility condition**					**<0.001**
Good	Reference				
Fair	0.6	0.3	1.8	0.9, 3.4	0.074
Poor	2.0	0.4	7.6	3.5, 16.5	<0.001
**Incident included road rage**					
No	Reference				

Yes	-2.3	0.4	0.1	0.04, 0.2	**<0.001**
**Did driver lose control of vehicle?**					
No	Reference				
Yes	1.1	0.3	2.9	1.7, 4.8	**<0.001**
**Was vehicle driver exceeding the speed limit?**					
No	Reference				
Yes	0.2	0.2	1.2	0.8, 1.9	**0.337**
**Did the vehicle driver pass too close?**					
No	Reference				
Yes	-1.0	0.2	0.4	0.2, 0.6	**<0.001**
**Vehicle speed and passing distance**					**<0.001**
Speeding & passing too close	0.7	0.3	2.0	1.2, 3.6	0.013
Speeding only	0.6	0.6	1.9	0.6, 5.5	0.256
Passing too close only	Reference				
Neither speeding nor passing too close	1.7	0.3	5.5	3.0, 9.9	<0.001
**Did the rider/handler lose control of the horse?**					
No	Reference				
Yes	0.2	0.2	1.2	0.8, 1.9	**0.411**
**Details of the main horse involved in the incident**					
**Horse age in years (continuous)**	-0.01	0.02	0.99	0.9, 1.0	**0.523**
**Horse age category in quartiles**					**0.113**
up to 7 years	0.1	0.3	1.1	0.6, 1.9	0.836
8-11 years	-0.4	0.3	0.6	0.3, 1.2	0.166
12-15 years	-0.6	0.4	0.5	0.3, 1.1	0.086
>15 years	Reference				
**Frequency of road use**					**<0.001**
More than once/week	Reference				
Weekly	-0.99	0.4	0.4	0.2, 0.8	0.014
Monthly	0.3	0.6	1.4	0.4, 4.5	0.58
Other	1.7	0.3	5.3	2.7, 10.5	<0.001
**Horse use**					**<0.001**
Ridden	Reference				
Horse-drawn vehicle	1.5	0.3	4.7	2.4, 9.0	<0.001
Leading	-0.3	0.5	0.7	0.3, 1.9	0.505
Loose	4.6	0.3	100.0	51.4, 194.5	<0.001
**Did horse fall?**					
No	Reference				
Yes	3.0	0.2	19.5	12.9, 29.5	**<0.001**

**Collision between the horse, rider/handler or horse-drawn vehicle and driver of vehicle**					
No	Reference				
Yes	5.2	0.6	188.2	59.7, 592.9	**<0.001**
**Details of the main rider/handler involved in the incident**					
**Rider/handler experience on the road**					**0.180**
0-5 years	0.7	0.4	2.1	0.9, 4.7	0.072
6-10 years	0.1	0.4	1.1	0.5, 2.6	0.74
11-15 years	0.6	0.3	1.7	0.9, 3.3	0.081
15 years+	Reference				
**Rider/handler age in years (continuous)**	-0.01	0.008	0.99	0.97, 1.0	**0.221**
**Rider/handler age category in quartiles**					**0.096**
up to 27 years	0.3	0.3	1.3	0.7, 2.3	0.394
28-40 years	-0.4	0.3	0.7	0.3, 1.3	0.262
41-50 years	-0.4	0.4	0.6	0.3, 1.3	0.218
>50 years	Reference				
**Rider/handler gender**					**<0.001**
Female	Reference				
Male	1.6	0.3	5.0	3.0, 8.3	
Unknown	2.3	0.2	9.7	6.3, 14.7	
**Severity of injury to rider/handler**					**<0.001**
None	Reference				
Mild	1.5	0.3	4.5	2.6, 7.8	<0.001
Moderate	2.2	0.3	9.0	5.5, 14.9	<0.001
Severe	3.3	0.3	26.5	15.5, 45.3	<0.001
Fatal	3.6	0.6	36.3	12.2, 108.3	<0.001
**High visibility clothing worn by the rider/handler or horse?**					
No	Reference				**<0.001**
Yes	-2.7	0.2	0.07	0.05, 0.1	
